# Tumor infiltrating lymphocytes: an intriguing player in the survival of colorectal cancer patients

**DOI:** 10.1186/1471-2172-11-19

**Published:** 2010-04-12

**Authors:** Vanessa Deschoolmeester, Marc Baay, Eric Van Marck, Joost Weyler, Peter Vermeulen, Filip Lardon, Jan B Vermorken

**Affiliations:** 1 Laboratory of Cancer Research and Clinical Oncology, Department of Medical Oncology, University of Antwerp (UA/UZA), Wilrijk, Belgium; 2 Department of Pathology, University Hospital Antwerp (UZA), Edegem, Belgium; 3 Department of Epidemiology and Social Medicine, University Antwerp, Wilrijk, Belgium; 4 Translational Cancer Research Group, Oncology Center, General Hospital St Augustinus, Wilrijk, Belgium

## Abstract

**Background:**

There is growing evidence that both local and systemic inflammatory responses play an important role in the progression of a variety of solid tumors. Colorectal cancer results from the cumulative effect of sequential genetic alterations, leading to the expression of tumor associated antigens possibly inducing a cellular anti-tumor immune response. It is well recognized that cytotoxic lymphocytes constitute one of the most important effector mechanisms of anti-tumor-immunity. However, their potential prognostic influence in colorectal cancer remains controversial. Aim of the study was to examine infiltration of CD3^+ ^and CD8^+ ^lymphocytes in colorectal cancer and their prognostic potential.

Two-hundred-fifteen colorectal cancer cases, previously analyzed for microsatellite instability (MSI), were selected for immunohistochemical detection of CD3^+^, CD8^+ ^infiltration and the expression of granzyme B. Prognostic relevance was assessed by survival analysis.

**Results:**

Strong correlations were found between the infiltration of lymphocytes and several clinicopathological variables. Survival analysis revealed that intra-epithelial infiltration of CD3^+ ^and CD8^+ ^T lymphocytes and stromal infiltration of CD3^+ ^lymphocytes had a major impact on the patients' overall survival in the univariate analysis, however independent of their association with MSI-status. In addition, it was also demonstrated that there was an important disease specific survival advantage for patients with microsatellite stable (MSS) tumors containing intraepithelial CD8^+ ^tumor infiltrating lymphocytes. When samples were analyzed for colon cancer and rectal cancer separately, the results of the overall population were confirmed in colon cancer only. When entered into a multiple Cox regression analysis adjusting for other possible important confounding factors, the strong impact of lymphocyte infiltration on overall survival was not maintained. Only early stage and young age (borderline significant for overall population only) were associated with a better overall survival (early disease with disease-free survival also).

**Conclusions:**

In conclusion our results suggest a role for infiltrating CD3^+ ^and CD8^+ ^T lymphocytes in colorectal cancer whereby tumor infiltration could reflect a general principle of antitumor immunity, irrespective of the MSI-status.

## Background

Colorectal cancer belongs to the most common malignancies in the Western World [1]. The treatment of choice remains surgical resection. For patients who undergo successful surgery for colorectal cancer, adjuvant chemotherapy and/or radiotherapy is recommended in cases of high risk stage II and III disease [2,3]. Although the introduction of new chemotherapeutic agents improved the prognosis of colorectal cancer over the past decades, the outlook for most patients still remains relative poor [1,3]. Therefore, new treatment options, besides the standard therapies, seem warranted to further improve survival of patients with colorectal cancer [4], especially for stage II disease [5,6]. New approaches focus on immunotherapeutic strategies as there is growing evidence in recent years supporting the existence of cancer immunosurveillance [4].

It has been recognized that disease progression in cancer patients is not solely determined by the characteristics of the tumor but also by the host response. Indeed, there is growing evidence that both local and systemic inflammatory responses play an important role in the progression of a variety of solid tumors [7-10]. In addition, the interrelationship between both inflammatory responses might have an influence on the outcome of the disease [7-10]. Colorectal carcinogenesis is a multistep process, during which (epi)genetic alterations determine the transition from a normal to a malignant cell. Acquisition of these alterations requires, among others, destabilization of the genome. Several forms of genetic instability (microsatellite instability (MSI), chromosomal instability and epigenetic instability) are believed to be involved in the development of colorectal cancer. MSI can lead to the production of abnormal proteins and derived peptides that, by acting as neo-antigens [11], could induce an adaptive immune response effective in limiting tumor growth and/or spread [11-16]. Nevertheless, the antitumor immune response is complex, involving the interaction of several cell types and cell products of the adaptive as well as the innate immune system [7,17]. On the other hand, colorectal tumors are also capable of escaping immune surveillance using several strategies [18].

It is well recognized that cytotoxic T lymphocytes (CD8^+ ^T cells) constitute one of the most important effector mechanisms of anti-tumor immunity [17]. In order for CD8^+ ^T cells to recognize antigens, these need to be exposed on the tumor cells in association with the human leukocyte antigen (HLA) class I proteins. Upon encounter of a tumor cell antigen/HLA I complex for which their T cell receptor is specific, cytotoxic T lymphocytes clonally expand and subsequently differentiate. Part of the differentiation process into killer cells includes the formation of a large number of modified lysosomes loaded with perforin and several types of granzymes [19,20]. These activated cytotoxic T lymphocytes can mediate specific destruction of tumor cells by the release of these lytic components in case of direct cell-cell interaction. Perforin and enzymatic proteases (such as granzyme B) are released and cause cell death by disruption of the cell membrane and activation of the apoptotic pathway respectively [17,21]. In addition, other factors of the adaptive immune system play a role in the cancer immunosurveillance. CD4^+ ^T cells, which only respond to antigens presented by the HLA class II proteins expressed by antigen presenting cells (like dendritic cells), are important for antitumor immunity. Depending on the cytokine profile induced, CD4^+ ^T cells are subdivided in T helper-1 cells or T helper-2 cells. Importantly, T helper-1 cells are essential for the proliferation of cytotoxic T lymphocytes as these lymphocytes require T helper-1 effector cell produced interleukin-2 for their proliferation. Although it has been shown that CD4^+ ^T cells may be sufficient by themselves to eliminate tumor cells, it is more often the case that both CD4^+ ^and CD8^+ ^T cells are required for effective tumor cell elimination because most tumor cells only express HLA class I molecules. However, the induction of cytotoxic T lymphocytes responses takes time, leaving time for the tumor cells to escape the immune system. Therefore, natural killer cells from the innate immune system may also play an important role since these cells can lyse natural killer-sensitive tumor targets prior to antigen sensitization or clonal expansion. Given that natural killer cells are not HLA restricted, these cells bear the capacity to eliminate tumor cells that do not express HLA. In contrast, HLA complexes are recognized by inhibitory receptors on natural killer cells resulting in their inactivation. In addition, natural killer cells express several ligands of the tumor necrosis factor family and can induce apoptosis of malignant cell targets which are phagocytosed by dendritic cells and macrophages and processed for subsequent presentation to T cells. Furthermore, natural killer cells constitutively express the interleukin-2 receptor and are able to respond to interleukin-2 stimulation that results in augmented cytotoxic activity [19].

The potential influence of immune-cell infiltrates in various neoplasms, including colorectal cancer, on the prognosis of patients is investigated in several studies but remains inconclusive [4,22,23]. Pronounced lymphocyte infiltration in colorectal cancer has been described and is more marked in MSI-positive tumors [15,16,24-27]. Additionally, these tumors predominantly have a proximal location and are reported to run a better clinical course (as reviewed in [28,29]). Some groups found that stage III colorectal tumors with a high content of intratumoral lymphocytes had a more favorable clinical outcome [30,31]. In contrast, in a randomized trial it was demonstrated that active specific immunotherapy, using autologous tumor cells and Bacillus Calmette-Guérin, had a significant clinical benefit in the adjuvant treatment of stage II colon cancer only [32,33].

Since several studies [13,30,34] showed the presence of a cytotoxic antitumor immune response in subsets of colorectal cancer, the aim of this study was to examine infiltration of CD3^+ ^and CD8^+ ^lymphocytes in colorectal cancer according to the classification of Naito et al. [24]. Correlation with overall and disease-free survival was calculated in order to determine the potential prognostic role of cytotoxic T lymphocytes.

## Results

### Patient characteristics

Of the 215 colorectal cancer patients from whom tumor tissue could be obtained, most (but not all) clinical data could be retrieved. Most tumors were located in the distal part of the large bowel (63.3%) and most patients had a stage II or III disease. Further details on these patients are summarized in Table 1.

**Table 1:** Patient characteristics

Patient characteristics		Colon	Rectum	Overall population
Total no. of patients		141	64	215
Median age (years)		70 +/- 12	64 +/- 13	68 +/- 13
Sex				
	male	62 (44.0%)	40 (62.5%)	107 (49.8%)
	Female	79 (56.0%)	24 (37.5%)	106 (49.3%)
Location				
	proximal			77 (35.8%)
	distal			136 (63.3%)
Grade of differentiation				
	poor	17 (12.1%)	2 (3.1%)	19 (8.8%)
	moderate	39 (27.7%)	19 (29.7%)	63 (29.3%)
	well	83 (58.9%)	39 (60.9%)	126 (58.6%)
Stage				
	I	15 (10.6%)	15 (23.4%)	30 (14.0%)
	II	53 (37.6%)	19 (29.7%)	74 (34.4%)
	III	59 (41.8%)	18 (28.1%)	80 (37.2%)
	IV	13 (9.2%)	10 (15.6%)	26 (12.1%)
Therapy				
Neo-adjuvant	Yes	1 (0.7%)	16 (25.0%)	19 (8.8%)
	No	128 (90.8%)	44 (68.8%)	178 (82.8%)
Adjuvant	Yes	47(61.0%)	30 (45.9%)	81 (37.7%)
	No	86 (33.3%)	29 (45.3%)	119 (55.3%)
MSI status				
	MSI	24 (17.0%)	2 (3.1%)	27 (12.6%)
	MSS	117 (83.0%)	62 (96.6%)	188 (87.4%)

Clinical characteristics of patients analyzed for the overall population and for patients with colon cancer and rectal cancer separately (in 10 patients the actual location of the tumor was not specified; not all clinical characteristics were available for each patient) (MSI: microsatellite instability, MSS: microsatellite stability).

### Immunohistochemistry

To enhance the power of the study the prognostic relevance of lymphocyte infiltration was assessed based on the comparison of two levels of infiltration: nil and mild infiltration on the one hand and moderate and severe infiltration on the other hand.

**Figure 1:**
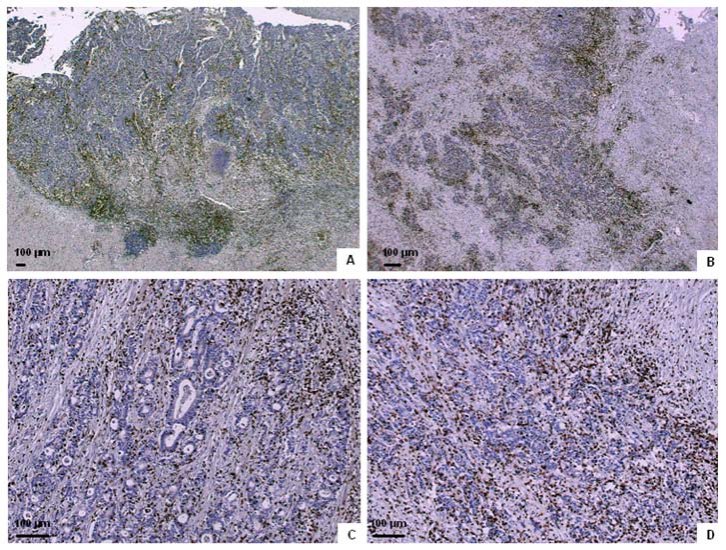
**Immunohistochemical staining of lymphocyte infiltration in colorectal cancer**. Examples of lymphocyte infiltration in colorectal tumors. **A **and **C**: CD8^+ ^T cells infiltrating the invasive margin (**A**), the stroma (**A **and **C**) and in the cancer cell nests (**C**). **B **and **D**: CD3^+ ^cells infiltrating the invasive margin (**B**), the stroma (**B **and **D**) and in the cancer cell nests (**D**).

**Figure 2:**
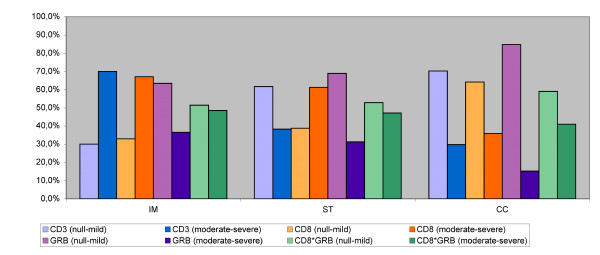
**Semiquantitative scoring of inflammatory infiltration**. Semiquantitative scoring of inflammatory infiltration of the three different tumor regions, based on two levels of infiltration (null-mild versus moderate-severe). (IM: invasive margin, ST: stroma, CC: cancer cell nests, GRB: granzyme B, CD8*GRB: CD8 T cells expressing GRB).

Semi-quantitative scoring of inflammatory infiltration of the three different tumor regions is shown in Table 2 and Figure 1. It is shown that infiltration of CD3^+ ^T cells is most abundant in the invasive margin while infiltration of CD8^+ ^cytotoxic T lymphocytes is strongest in the invasive margin and the stroma which means that in this study population, most T lymphocytes were found in the invasive margin but the cytotoxic T lymphocytes were also abundant in the stroma close to the tumor cells. The expression of granzyme B is much sparser. Approximately 50% of CD8^+ ^T cells express their associated cytotoxic molecule granzyme B moderately/severely (see also CD8*GRB in Table 2 and Figure 2).

**Table 2:** Semiquantitative scoring of inflammatory infiltration in colorectal cancer.

	infiltration	IM	ST	CC
CD3	0	30.0%	61.7%	70.2%
	1	70.0%	38.3%	29.8%
CD8	0	32.9%	38.8%	64.1%
	1	67.1%	61.2%	35.9%
GRB	0	63.4%	68.9%	84.7%
	1	36.6%	31.3%	15.3%
CD8*GRB	0	51.4%	52.9%	59.0%
	1	48.6%	47.1%	41.0%

Semiquantitative scoring of inflammatory infiltration of the three different tumor regions, based on two levels of infiltration. (%: percentage of samples with particular scoring, 0: null-mild infiltration, 1: moderate-severe infiltration, IM: invasive margin, ST: stroma, CC: cancer cell nests, GRB: granzyme B, CD8*GRB: CD8 T cells expressing GRB).

Strong associations were found between all infiltrating lymphocytes in the different tumor regions, except for CD3^+ ^T cells and granzyme B expression in the invasive margin and the stroma. In addition, several strong correlations were found between the infiltration of lymphocytes and clinicopathological variables, such as poorly differentiated tumors (CD8^+ ^in stroma and cancer cells, CD3^+ ^and granzyme B in invasive margin), distal location (CD3^+ ^in cancer cells and granzyme B in invasive margin), early stage (CD8^+ ^in cancer cells, CD3^+ ^in invasive margin and cancer cells and granzyme B in cancer cells) and the absence of treatment (CD8^+ ^in all areas CD3^+ ^and granzyme B in invasive margin and stroma).

### Correlation of MSI and lymphocyte infiltration

As previously mentioned, all samples had been typed for MSI using the mononucleotide multiplex system [35]. Twenty-seven out of 215 colorectal samples (12.6%) showed a high level of MSI (MSI-H) and 188 out of 215 showed microsatellite stability (MSS).

In this study population, sporadic MSI-H tumors were associated with proximal location (p < 0.001), poor differentiation (p < 0.001), female gender (p = 0.018) and there was a trend towards early stage (p = 0.077). A higher infiltration of CD8^+ ^and CD3^+ ^lymphocytes was noted in all examined areas of MSI-H tumors compared to MSS tumors. Granzyme B expression was considerably higher within the cancer cell nests of MSI-H tumors. In addition, logistic regression showed that MSI (HR: 5.505, p < 0.001) and stage (HR: 0.582, p = 0.006) were the major predictors of the presence of activated intraepithelial CD8^+ ^T lymphocytes whereas grading, age, location and gender did not show a major impact.

### Correlations with survival

Follow up for overall survival was available for 209 colorectal cancer patients. At the end of the observation period, 100 patients (47.8%) were deceased and all deaths were cancer related. Follow up for disease-free survival was available for 193 colorectal cancer patients and 57 patients (29.5%) experienced a recurrence of the tumor. The median follow up for overall and disease-free survival was 5.2 and 4.9 years respectively.

**Figure 3:**
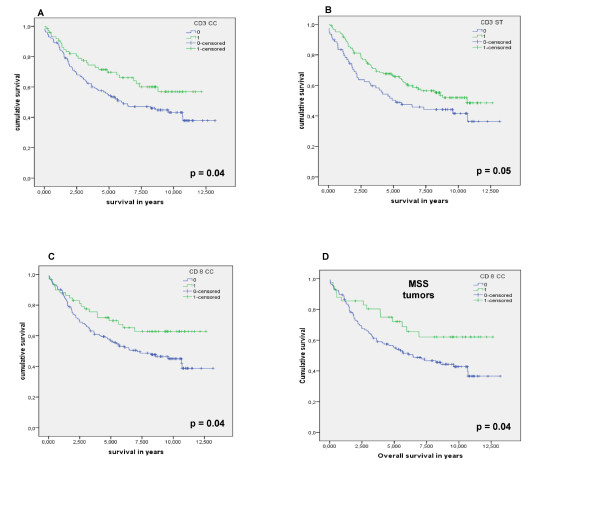
**Kaplan-Meier survival curves for infiltrating lymphocytes in colorectal cancer**. Kaplan-Meier curves for overall survival among colorectal cancer patients according to (**A**) the infiltration of CD3^+ ^lymphocytes in the cancer cell nests and (**B**) the stroma, (**C**) according to the infiltration of CD8^+ ^lymphocytes in the cancer cell nests and (**D**) according to the infiltration of CD8^+ ^lymphocytes in the cancer cell nests of MSS tumors (IM: invasive margin, ST: stroma, CC: cancer cell nests, MSS: microsatellite stable).

In univariate survival analysis, patients with MSI-H tumors showed a slightly longer overall survival than patients with MSS tumors, however, significance was not reached (HR: 0.55, 95%CI: 0.26-1.131). As expected, tumor stage (HR: 2.20, 95%CI: 1.72-2.83) and age (HR: 2.29, 95%CI: 1.51-3.46) had the most impact on overall survival while only early stage (HR: 2.32, 95%CI: 1.60-3.37) was correlated with a better disease-free survival. Furthermore, univariate analysis also showed that female gender (HR: 1.51, 95%CI: 1.02-2.26), high differentiation grade (HR: 0.83, 95%CI: 0.70-0.99) and distal location (HR: 0.65, 95%CI: 0.44-0.98) had an important influence on longer overall survival. In addition, infiltration of CD3^+ ^lymphocytes in stroma and in cancer cell nests and infiltration of CD8^+ ^T lymphocytes in cancer cell nests had an important impact on overall survival. Furthermore, MSS colorectal cancers with intra-tumoral infiltration of CD8^+ ^T lymphocytes had a better overall survival compared to MSI-positive colorectal tumors (figure 3).

Multiple Cox regression analysis was used to verify whether the investigated variables concerning T cells are valid when taking into consideration potential confounding cofactors (Table 3). The positive impact of tumor infiltrating lymphocytes on overall survival was lost and only early stage (p < 0.001) and young age (p = 0.04) were independently associated with a better overall survival. For disease-free survival, early stage (p < 0.001) was the only independent factor associated with a better outcome (table 3).

**Table 3:** Survival analysis using a Cox regression model.

	Overall survival				Disease free survival			
	Cox Regression				Cox Regression			
	HR	95%CI		p-value	HR	95%CI		p-value
MSI	0.38	0.12	1.18	0.09	0.33	0.09	1.27	0.11
Age	1.04	1.00	1.07	0.04	0.97	0.92	1.01	0.14
Gender	1.41	0.79	2.53	0.25	1.45	0.68	3.13	0.34
Localization	0.71	0.38	1.31	0.27	1.16	0.47	2.84	0.75
Grade of differentiation	0.70	0.43	1.13	0.15	0.53	0.34	1.09	0.05
stage	2.37	1.58	3.56	< 0.001	2.70	1.51	4.83	< 0.001
adjuvant treatment	0.70	0.29	1.68	0.43	1.03	0.36	2.97	0.96
CD8IM	1.24	0.56	2.75	0.59	0.82	0.29	2.36	0.71
CD8ST	0.89	0.38	2.10	0.79	1.95	0.66	5.76	0.22
CD8CC	2.06	0.67	6.39	0.21	0.68	0.16	2.88	0.60
CD3IM	0.97	0.40	2.32	0.94	0.86	0.27	2.75	0.80
CD3ST	1.05	0.51	2.19	0.89	0.66	0.24	1.85	0.43
CD3CC	0.54	0.18	1.59	0.26	1.61	0.44	5.95	0.48
GRBIM	0.59	0.24	1.44	0.24	1.14	0.37	3.52	0.81
GRBST	0.86	0.37	2.00	0.72	0.53	0.17	1.65	0.27
GRBCC	1.18	0.45	3.13	0.74	2.18	0.62	7.61	0.22

Survival analysis of colorectal cancer patients using a multiple Cox regression model. (HR: hazard ratio, CI: confidence interval, IM: invasive margin, ST: stroma, CC: cancer cell nests, GRB: granzyme B, MSI: microsatellite instability).

**Figure 4:**
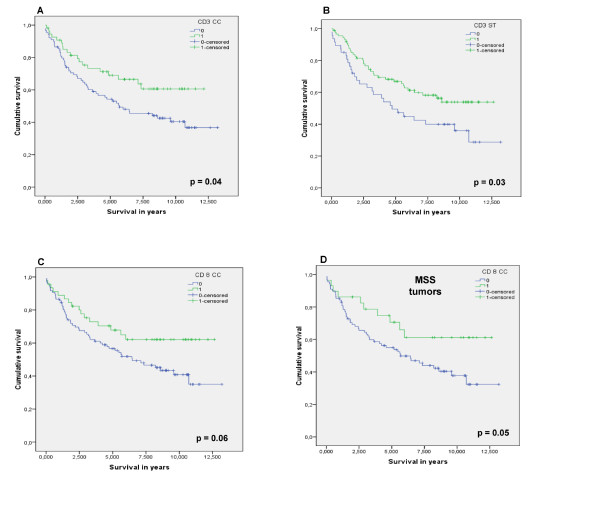
**Kaplan-Meier survival curves for infiltrating lymphocytes in colon cancer only**. Kaplan-Meier curves for overall survival among colon cancer patients according to (**A**) the infiltration of CD3^+ ^lymphocytes in the cancer cell nests and (**B**) the stroma, (**C**) according to the infiltration of CD8^+ ^lymphocytes in the cancer cell nests and (**D**) according to the infiltration of CD8^+ ^lymphocytes in the cancer cell nests of MSS tumors (IM: invasive margin, ST: stroma, CC: cancer cell nests, MSS: microsatellite stable).

When samples were analyzed for colon cancer and rectal cancer separately using univariate analysis, infiltration of CD3^+ ^lymphocytes in stroma and in cancer cell nests and infiltration of CD8^+ ^T lymphocytes in cancer cell nests had a major impact on overall survival in colon cancer but not in rectal cancer (see further below). In addition, MSS colon cancers with intra-tumoral infiltration of CD8^+ ^T lymphocytes had a considerably better overall survival compared to MSI-positive colon cancers (figure 4). Again, in the multiple Cox regression analysis infiltration of lymphocytes was not retained as an independent prognostic factor. The most important confounding factor was early stage which was strongly associated with a better overall and disease-free survival. In contrast, as mentioned, infiltration of lymphocytes in rectal cancer had no influence on survival in the univariate analysis. The administration of pre-operative radiotherapy in rectal cancer might destroy the immune cells present in the tumor prior to surgical resection. However, when rectal cancer patients who received pre-operative radiotherapy were removed from the analysis, the associations between the infiltration of CD3^+ ^lymphocytes in stroma and in cancer cell nests and infiltration of CD8^+ ^T lymphocytes in cancer cell nests and overall survival were only slightly stronger in the overall population. Once more these associations could not be confirmed in the Cox regression analysis. In addition, infiltration of lymphocytes in rectal cancer without pre-operative radiotherapy still had no influence on survival.

## Discussion and Conclusions

Solid tumors are not just composed of malignant cells, but are complex microcosms of many cell types, including a wide range of hematopoietic cells [36]. Anti-tumor immune responses may be one of the most important weapons in the arsenal against cancer [37]. Galon et al. [38,39] suggested that once human colorectal tumors become clinically detectable, the adaptive immune response plays a role in preventing tumor recurrence and metastasis. Intratumoral T cells could modify tumor stroma or tumor cells in ways that attenuate the metastatic potential of tumor cells [40,41]. However, it is still unclear if the presence of tumor infiltrating lymphocytes represents the result of an inflammatory response that facilitates either tumor progression or a protective host response against cancer [4].

The present study confirms that tumor infiltrating lymphocytes are present in colorectal cancer and, as expected, a strong correlation was found between CD3^+ ^and CD8^+ ^T cells. Additionally, a strong association was also seen between the presence of CD8^+ ^T lymphocytes and the expression of their associated cytotoxic molecule granzyme B in all examined areas of the tumor. Granzyme B expression was much sparser which can be explained by the fact that only those CD8^+ ^T lymphocytes which are cytolytic (about 50% in this study) will express their associated cytotoxic molecule granzyme B. When exposed to dysfunctional cells, cytotoxic T lymphocytes will release granzyme B and will eventually induce apoptosis of the proximal tumor cells bearing the appropriate antigen determinants [7,17]. This might hold promise in the field of immunotherapy [16,42]. Unfortunately, at this time we were unable to investigate T cell surface activation markers or the apoptosis status of the infiltrated tumor cells in order to confirm the lytic function of these infiltrating T cells.

As found in several other studies [7,39,43-48], the presence of a pronounced lymphocytic infiltration within the tumor is associated with improved survival in the current study. Specifically, CD3^+ ^and CD8^+ ^T lymphocytes within cancer cell nests of colorectal cancer and of CD3^+ ^in the colorectal cancer stroma had a major impact on the patients' overall survival by univariate analysis in the overall population. When colon and rectal tumors were investigated separately, the results of the overall population were confirmed in the group of colon cancer patients but not in the group of the rectal cancer patients, even when the rectal cancer patients receiving pre-operative radiotherapy were excluded from the analysis.

The improved survival associated with infiltration of lymphocytes may be the result of an effective suppression of micrometastases in distant organs or near the primary site. In other words, the number of CD8^+ ^T cells within the primary tumor might be a good indicator of the presence of a systemic immunosurveillance mechanism [40,41]. In addition, tumor cells can secrete substances in the stroma, which might be recognized by the immune system to destroy the tumor.

In accordance to Pages et al. [47], Ropponen et al [44] and Koch et al [4], but in contrast to Ling et al. [49], stage was a major determinant of infiltration of activated cytotoxic intraepithelial lymphocytes (as shown by multivariate analysis) with a higher infiltration in earlier stages (HR: 0.582, p = 0.006). This might indicate that immune reactions may be more prominent in early stages of disease and might have a stage specific influence on survival [31,50], possibly irrespective of the MSI-status. There might be a protective local immune response in these earlier tumor stages, preventing further tumor growth and spread. However, this hypothesis has to be substantiated by further analysis of activation status of tumor infiltrating lymphocytes in patients with early tumor stages. Alternatively, host immune response against cancer cells may decrease with increasing tumor growth [4].

Several groups found stage specific beneficial effects of infiltrating lymphocytes on clinical outcome [30-33]. Guidoboni et al. described a correlation between the presence of cytotoxic T lymphocytes and improved survival in stage II and III colorectal tumors irrespective of the MSI-status [30]. These results were confirmed by Prall et al. [31] for stage III disease. However, when MSI-status was taken into account, the correlation with survival was most pronounced when both MSI-H and intratumoral-activated cytotoxic T lymphocytes were present in stage III tumors [30].

In this study, stage specific survival analysis did not show a major influence of tumor infiltration on overall survival. However, given the relative small number of cases per stage group, such an influence might be difficult to detect. Nevertheless, this could be of interest since we previously showed a significant beneficial effect of active specific immunotherapy in adjuvant treatment of patients with stage II colon cancer only [32].

Accumulating evidence indicates that most MSI-positive colorectal tumors are characterized by the presence of a pronounced intratumoral inflammatory reaction, the nature of which, however, is still poorly understood [30,51]. Within these tumors, tumor infiltrating lymphocytes have been identified as predominantly activated CD8^+ ^T cells. The presence of these cytotoxic T lymphocytes has been attributed to the inherently greater production of abnormal peptides as a result of unreliable DNA repair in MSI-positive tumors [37]. In the current study, among the different clinicopathological parameters analyzed, MSI was strongly correlated with a high presence of activated cytotoxic intraepithelial lymphocytes. Nevertheless, survival analysis by MSI-status revealed that, despite the high infiltration of lymphocytes in MSI-positive colorectal tumors, no association was found with a favorable prognosis within this subset. These data are in agreement with some studies [31,37,50,52-57], but they are in conflict with a number of other reports [13,24-27,30,34,58] in which a predominant presence of intra-tumor cell infiltrating lymphocytes in MSI-H cancers was significantly correlated with a better prognosis. In accordance to Baker et al. [37], MSS colorectal tumors with infiltration of CD8^+ ^tumor infiltrating lymphocytes showed a better overall survival. One explanation might be that the functions of tumor infiltrating lymphocytes differ between these subgroups. It is possible that the high prevalence of recruitment and retention of tumor infiltrating lymphocytes in MSI-positive colorectal tumors is inherent to the unique aspect of the tumor biology, supporting the hypothesis that the genetic instability of MSI-positive colorectal tumors may lead to the production of a greater number of tumor specific antigens that trigger the immune system [7,11-16,27,30]. As described previously [59], direct contact between tumor cells, presenting these aberrant peptides, and leucocytes plays a crucial role in the immune reaction. However, the concept of cross-priming in which antigen presenting cells pick up antigens released by dead tumor cells and subsequently present them to T cells may also be important in vivo [42].

On the other hand, MSI-positive tumors can undergo immuno-editing, which dictates that tumors reaching the stage of clinical detection have been shaped antigenically by the initial immune responses mounted against them to a point where they are no longer recognized as foreign to the body. In MSS tumors, which arise in a much less immunologically stimulating environment, the tumors are less likely to have adapted antigenically and may thus be more sensitive to late stage immune attack. Thus, even though MSI-positive tumors produce more tumor specific antigens that might trigger the immune system, the elevated levels of these antigens in combination with the lack of appropriate costimulatory molecules on the tumor cells may generate a microenvironment which leads to a state of tumor infiltrating lymphocyte anergy, thereby preempting any beneficial effect on survival [12,37]. In addition, the expression of HLA class I proteins, presenting tumor associated antigens on the tumor cell surface, is considered a prerequisite for an effective T cell immune response [60]. It has been described that genetically unstable tumors are often HLA class I negative and might escape T-cell mediated immune killing. However, in the absence of any surface HLA class I these tumors would be susceptible to a natural killer cell attack. In contrast, those tumors that downregulate specific HLA class I alleles may avoid both T cell and natural killer cell activation [20]. Therefore, Speetjens et al. [60] hypothesized that both oncogenic pathway and HLA class I expression might dictate clinical tumor progression. Furthermore, insufficient evidence exists to recommend routine use of biological factors as MSI for either determining prognosis or predicting response to therapy in colorectal cancer patients [61].

When entered into a multiple Cox regression analysis adjusting for other possible important confounding factors, only early stage and young age (borderline significant for overall population only) were associated with a better overall survival (early stage also with disease-free survival). The beneficial effect of tumor infiltrating lymphocytes in colorectal cancer could not be confirmed in the Cox regression survival analysis.

In summary, this study confirms that tumor infiltrating lymphocytes are indeed important clinical and prognostic indicators in colorectal cancer, in univariate analysis, irrespective of the MSI-status. Therefore, it was considered that tumor infiltration could reflect a general principle of antitumor immunity as mentioned by Prall et al. [31]. In addition, in accordance with Baker et al., we also demonstrated an important disease-specific survival advantage for patients with MSS tumors containing intraepithelial CD8^+ ^tumor infiltrating lymphocytes [37]. Although no stage specific survival difference of tumor infiltrating lymphocytes was noted in colorectal cancer, it was demonstrated that stage was also a major determinant of the presence of tumor infiltrating lymphocytes in colorectal cancer, with a higher infiltration in earlier stages.

We would like to note that the tumor immune response does not only depend on infiltration and activation of T lymphocytes. Therefore, the minute balanced association of all infiltrating cells, their capability to synthesize and release tumor-modifying substances, and their content of presynthesized, granule-associated bioactive substances deserves intensive study. By doing so, new biological tumor-host responses may be disclosed, leading to the development of new approaches to the treatment of malignant disease [45].

## Methods

### Tissue samples

Tumor DNA was obtained from 215 patients with surgically resected colorectal cancer, treated between 1995 and 2003, from the Antwerp University Hospital and the St. Augustinus Hospital Antwerp. The study was approved by the local Ethics Committee of the University of Antwerp and was conducted in accordance with the ethical principles stated in the most recent version of the Declaration of Helsinki.

### Immunohistochemistry

Five μm-thick sections were prepared from formalin-fixed paraffin-embedded tissue for IHC. Sections were deparaffinized in toluene, dehydrated and subjected to heat antigen retrieval by Tris EDTA buffer (pH 9) in a heating bath for 30 min. at 95 (± 1)°C.

Sections were subsequently stained using the Dako Autostainer Plus system (DAKO, DakoCytomation, Glostrup, Denmark). Endogenous peroxidase activity was quenched by incubating the slides in peroxidase block EnVision Plus (DAKO EnVision™ kit, DakoCytomation, Glostrup, Denmark) for 10 minutes. Incubation with primary monoclonal antibodies against: CD3 (clone SPF7, diluted 1:150, Neo-Markers, CA, USA), CD8 (clone 1A5, diluted 1:20, Novacastra Laboratories Ltd, Newcastle upon Tyne, UK) and Granzyme B (clone GRB7, diluted 1:25, DAKO, DakoCytomation, Glostrup, Denmark) was performed for 30 minutes at room temperature. Sites of binding were detected using the Envision dual link detection system (DAKO EnVision™ kit, DakoCytomation, Glostrup, Denmark) with 3,3'-diaminobenzidine (DAB^+^) as chromogen according to the manufacturer's instructions. The sections were counterstained with haematoxylin, dehydrated, cleared and mounted.

### Classification and quantification of lymphocytes

Inflammatory infiltration by CD3^+ ^(early T cell marker), CD8^+^(cytotoxic T cell marker) T lymphocytes and the expression of Granzyme B (marker for cytotoxic cells after cell activation) were immunohistochemically scored in three regions of the tumor as described by Naito et al. [24]: a) those distributed along the invasive margin of the tumor, b) those infiltrated in cancer stroma and c) those infiltrated in the tumor cell nests.

Each entire tumor section was evaluated for infiltrating cells by using a × 10 objective lens. The number of lymphocytes infiltrating the tumor cell nests was counted in five visual fields, selected for most abundant distribution under a light microscope at × 400 magnification, corresponding to a total area of 1.19 mm^2^. The degree of infiltration was classified as follows: nil: 0, mild: 1-19, moderate: 20-49 and severe: ≥ 50 positive cells [24].

For all other regions of the tumor, immune reactivity was scored semi-quantitatively (nil, mild, moderate and severe) as described by Naito et al., [24] Scoring was performed by two independent observers, if no consensus could be reached a third observer was consulted.

### MSI analysis

All cases had been previously analyzed for MSI-status [62]. After manual microdissection of formalin-fixed, paraffin embedded tissue blocks, DNA was isolated as described previously [63]. MSI analysis was performed using the mononucleotide multiplex system as described earlier [35]. In short, the sense primers were chemically labeled at the 5' end with FAM™ fluorescent dyes. PCR was carried out in a final volume of 25 μl containing 200 μmol/L dNTPs (MBI Fermentas, St. Leon-Rot, Germany), 500 nM of each sense and antisense primer (Eurogentec, Seraing, Belgium), 1 × PCR buffer (60 mM Tris SO_4 _(pH 8.9), 18 mM (NH_4_)SO_4 _and 2 mM MgSO_4_) and 1 unit Discoverase dHPLC DNA polymerase (Invitrogen, Merelbeke, Belgium). Fluorescent PCR products were analyzed by capillary electrophoresis using an ABI 3100 Genetic Analyzer (Applied Biosystems, Lennik, Belgium) and Genemapper Software 3.7.

### Statistical analysis

Prognostic relevance of lymphocyte infiltration was assessed by survival analysis.

The index date for survival time calculation was defined as the date of surgery for colorectal cancer. The months of observation (= overall survival time) were calculated from the index date to the date of last information/death. For disease free survival time the months of observation were calculated from the index date to the first date of progressive disease. Survival curves were determined by using the Kaplan-Meier method and were analyzed by using the log-rank test.

Possible associations between MSI-status and the immunophenotypic markers and clinicopathological parameters of colorectal cancers were investigated using the χ^2^-test or Fisher's exact test (when numbers expected per cell are less than 5) for categorical variables and using Student t-test (for unpaired comparisons based on numerical variables for which the assumption of a normal distribution was appropriate (i.e. Kolmogorov-Smirnoff test p > 0.02)) or its nonparametric alternative Mann-Whitney U test (for unpaired comparisons based on numerical variables for which the assumption of a normal distribution was not appropriate) for continuous variables. In order to assess the independent prognostic contribution of the cytotoxic T lymphocytes, a multiple Cox regression analysis was conducted. All analyses were conducted using SPSS (version 16.0). Significance for all statistics was recorded if p < 0.05 (two-tailed).

## Authors' contributions

VD designed the study, carried out the MSI analysis, the IHC scoring, the statistical analysis and the interpretation of the results and drafted the manuscript. MB participated in the study design, carried out the IHC scoring and helped to draft the manuscript. EVM participated in the IHC and supervised the scoring and revised the manuscript. JW supervised the statistical analysis and revised the manuscript. PV provided tissue samples and coordinated sifting through the medical files. FL and JBV participated in the design and coordination of the study and helped to draft the manuscript. All authors read and approved the final manuscript.
